# Supplementary material for: Coexisting Conditions Modifying Phenotypes of Patients with 22q11.2 Deletion Syndrome
Source: Genes (Basel). 2023 Mar 9;14(3):680. doi: 10.3390/genes14030680 (PMC10048180; doi:10.3390/genes14030680)
Supplement: Supplementary file 1 [file genes-14-00680-s001.zip › Supplementary Table S1.pdf]

Table S1. On the basis of physical examination for every patient, a detailed form with over 230 clinical features was completed.

Patient name:

Date of birth:

Sex:

Sample ID:

Birth: …… Hbd ☐ vaginal ☐ CS, reason:………………….

	NO	YES	COMMENTS
**Cardiovascular system defects**			
Tetralogy of Fallot	No ☐	Yes☐	
Ventricular septal defect - VSD	No ☐	Yes ☐	☐ perimembranous<br>☐ inlet<br>☐ subarterial<br>☐ muscular
Interrupted aortic arch - IAA	No ☐	Yes ☐	☐ A ☐ B ☐ C
Persistent truncus arteriosus, PTA	No ☐	Yes ☐	
Vascular ring - VR	No ☐	Yes ☐	
Pulmonary atresia - PA	No ☐	Yes ☐	
Bicuspid aortic valve - BAV	No ☐	Yes ☐	
Atrial septal defect – ASD	No ☐	Yes ☐	
Conotruncal cardiac anomaly	No ☐	Yes ☐	
Aberrant subclavian	No ☐	Yes ☐	
Cyanosis	No ☐	Yes ☐	
other			
**Immune system insufficiency**			
Defects in humoral immunity	No ☐	Yes ☐	
IgA deficiency	No ☐	Yes ☐	
Recurrent infections	No ☐	Yes ☐	
Acute otitis media	No ☐	Yes ☐	
Chronic sinusitis	No ☐	Yes ☐	
T-cell lymphopenia	No ☐	Yes ☐	
Delay IgG production	No ☐	Yes ☐	
Thymic aplasia with absent T cells	No ☐	Yes ☐	
Autoimmune diseases	No ☐	Yes ☐	☐ Juvenile rheumatoid arthritis<br>☐ Vitiligo<br>☐ Graves' disease<br>☐ Thrombocytopenia<br>☐ Haemolytic anemia<br>☐ Neutropenia

other			
**Palatal defects**			
Submucosal cleft palate	No ☐	Yes ☐	
Cleft palate	No ☐	Yes ☐	☐ unilateral<br>☐ bilateral
Cleft lip	No ☐	Yes ☐	☐ unilateral<br>☐ bilateral
Uvula bifida	No ☐	Yes ☐	
other			
**Velopharyngeal insufficiency (VPI)**			
Hypernasal speech	No ☐	Yes ☐	
Increased nasal resonance	No ☐	Yes ☐	
Nasal regurgitation	No ☐	Yes ☐	
Pronunciation defects	No ☐	Yes ☐	
Articulation problems	No ☐	Yes ☐	
Velopharyngeal insufficiency postadenoidectomy	No ☐	Yes ☐	
other			
**Laryngotracheoesophageal anomalies**			
Laryngeal web	No ☐	Yes ☐	
Laryngotracheal malacia	No ☐	Yes ☐	
Subglottic stenosis	No ☐	Yes ☐	
other			
**Neonatal hypocalcemia**	No ☐	Yes ☐	
**Short stature**	No ☐	Yes ☐	
**Endocrinological disorders**	No ☐	Yes ☐	☐ Thyroid hypoplasia<br>☐ Hypothyroidism<br>☐ Hyperthyroidism<br>☐ Growth hormone deficiency<br>☐ Hypoparathyroidism<br>☐ other
**Growth delay**	No ☐	Yes ☐	
**Macrocephaly**	No ☐	Yes ☐	
**Microcephaly**	No ☐	Yes ☐	
Asymmetric crying face	No ☐	Yes ☐	
**Dysmorphic features**			
Long face	No ☐	Yes ☐	
Asymmetric face	No ☐	Yes ☐	
Triangular face	No ☐	Yes ☐	
Square face	No ☐	Yes ☐	
Bitemporal narrowing	No ☐	Yes ☐	
Prominent forehead	No ☐	Yes ☐	

Synophrys	No ☐	Yes ☐		
Arched eyebrows	No ☐	Yes ☐		
Deep-set eyes	No ☐	Yes ☐		
Hypertelorism	No ☐	Yes ☐		
Hypotelorism	No ☐	Yes ☐		
Epicantic folds	No ☐	Yes ☐		
Ptosis	No ☐	Yes ☐		
Short palpebral fissures	No ☐	Yes ☐		
Narrow palpebral fissures	No ☐	Yes ☐		
Upslanting palpebral fissures	No ☐	Yes ☐		
Hooded eyelid	No ☐	Yes ☐		
Distichiasis	No ☐	Yes ☐		
Short upturned nose	No ☐	Yes ☐		
Broad nasal root/nose	No ☐	Yes ☐		
High nasal bridge	No ☐	Yes ☐		
Wide nasal bridge	No ☐	Yes ☐		
Bulbous nasal tip	No ☐	Yes ☐		
Hypoplastic alae nasi	No ☐	Yes ☐		
Smooth philtrum	No ☐	Yes ☐		
Short philtrum	No ☐	Yes ☐		
Nasal dimple	No ☐	Yes ☐		
Full lips	No ☐	Yes ☐		
Small mouth	No ☐	Yes ☐		
Thin upper lip	No ☐	Yes ☐		
Downturned mouth	No ☐	Yes ☐		
Micrognathia	No ☐	Yes ☐		
Retrognathia	No ☐	Yes ☐		
Pointed chin	No ☐	Yes ☐		
Malar flattening	No ☐	Yes ☐		
Mikrotia	No ☐	Yes ☐		
Low-set ears	No ☐	Yes ☐		
Posteriorly rotated ears	No ☐	Yes ☐		
Protruding ears	No ☐	Yes ☐		
Overfolded helices	No ☐	Yes ☐		
Squared off helices	No ☐	Yes ☐		
Cup ear	No ☐	Yes ☐		
Helix deformities	No ☐	Yes ☐		

Earlobe deformities	No ☐	Yes ☐		
Pre-auricular pits	No ☐	Yes ☐		
Pre-auricular tags	No ☐	Yes ☐		
other				
**Structural eye defects and ocular disorders**				
Astigmatism	No ☐	Yes ☐		
Hyperopia	No ☐	Yes ☐		
Myopia	No ☐	Yes ☐		
Strabismus	No ☐	Yes ☐		
Posterior embryotoxon	No ☐	Yes ☐		
Scleracornea	No ☐	Yes ☐		
Anophthalmia	No ☐	Yes ☐		
Coloboma	No ☐	Yes ☐		
Tortuous retinal vessels	No ☐	Yes ☐		
Cataract	No ☐	Yes ☐		
Deep iris crypts	No ☐	Yes ☐		
Small optic nerves	No ☐	Yes ☐		
Amblyopia	No ☐	Yes ☐		
other				
**Skeletal abnormalities**				
Scoliosis	No ☐	Yes ☐		
Cervical spine instability	No ☐	Yes ☐		
Long fingers	No ☐	Yes ☐		
Syndaktyly	No ☐	Yes ☐		
Preaxsial palm polydactyly	No ☐	Yes ☐		
Postaxial palm polydactyly	No ☐	Yes ☐		
Preaxial foot polydactyly	No ☐	Yes ☐		
Postaxial foot polydactyly	No ☐	Yes ☐		
Butterfly vertebrae	No ☐	Yes ☐		
Additional ribs	No ☐	Yes ☐		
Rib absence	No ☐	Yes ☐		
Hypoplastic scapula	No ☐	Yes ☐		
Craniosynostosis	No ☐	Yes ☐		
other				
**Muscoskeletal system problems**				
Hypotonia	No ☐	Yes ☐		
Ligamentous laxity	No ☐	Yes ☐		

Idiopathic leg pain	No ☐	Yes ☐	
Patella dislocation	No ☐	Yes ☐	
Joint swelling	No ☐	Yes ☐	
Early morning stiffnes	No ☐	Yes ☐	
other			
**Dental problems**			
Delayed dental eruption	No ☐	Yes ☐	
Enamel hypoplasia	No ☐	Yes ☐	
Chronic caries	No ☐	Yes ☐	
other			
**Structural ear defects and hearing disorders**			
Secondary hearing loss	No ☐	Yes ☐	
Conductive hearing loss	No ☐	Yes ☐	
Sensorineural hearing loss	No ☐	Yes ☐	
External auditory canal atresia	No ☐	Yes ☐	
Cochlear anomalies	No ☐	Yes ☐	
Eustachian tube anomalies	No ☐	Yes ☐	
other			
**Balance disorders**	No ☐	Yes ☐	
**Structural CNS anomalies**			
Polymycrogyria	No ☐	Yes ☐	
Meningomyelocele	No ☐	Yes ☐	
Ventriculomegaly	No ☐	Yes ☐	
Cerebellar hypoplasia	No ☐	Yes ☐	
Cerebellar atrophy	No ☐	Yes ☐	
Enlarged cisterna magna	No ☐	Yes ☐	
Tethered cord	No ☐	Yes ☐	
Enlarged sylvian fissures	No ☐	Yes ☐	
Spina bifida	No ☐	Yes ☐	
Polyhydramnios	No ☐	Yes ☐	
Encephalocele	No ☐	Yes ☐	
inne			
Unprovoked seizures	No ☐	Yes ☐	
**Genitourinary anomalies**			
Dysplastic kidneys	No ☐	Yes ☐	
Horseshoe kidney	No ☐	Yes ☐	

Multicystic kidney	No ☐	Yes ☐	
Duplex kidney	No ☐	Yes ☐	
Hypoplastic kidney	No ☐	Yes ☐	
Hydronephrosis	No ☐	Yes ☐	
Renal artery absence	No ☐	Yes ☐	
Bladder wall thickening	No ☐	Yes ☐	
Duplicated collecting system	No ☐	Yes ☐	
Urinary bladder aplasia	No ☐	Yes ☐	
Uterus aplasia	No ☐	Yes ☐	
Cryptorchidism	No ☐	Yes ☐	
Phimosis	No ☐	Yes ☐	
Hypospadias	No ☐	Yes ☐	
Inguinal hernia	No ☐	Yes ☐	
Vesicoureteral reflux	No ☐	Yes ☐	
Recurrent urinary tract infections	No ☐	Yes ☐	
Enuresis	No ☐	Yes ☐	
Bedwetting	No ☐	Yes ☐	
Frequent urination	No ☐	Yes ☐	
Renal tubular acidosis	No ☐	Yes ☐	
other			
**Gastrointestinal problems**			
Feeding and swallowing problems	No ☐	Yes ☐	
Nasopharyngeal reflux	No ☐	Yes ☐	
Prominence of the cricopharyngeal muscle	No ☐	Yes ☐	
Abnormal cricopharyngeal closure, and/or diverticulum	No ☐	Yes ☐	
Esophageal dysmotility	No ☐	Yes ☐	
Gastroesophageal reflux	No ☐	Yes ☐	
Chronic constipation	No ☐	Yes ☐	
Abdominal pain	No ☐	Yes ☐	
Vomiting	No ☐	Yes ☐	
Dysphagia	Nie ☐	Yes ☐	
other			
**Gastrointestinal anomalies**			
Intestinal malrotation with secondary intestinal torsion	No ☐	Yes ☐	
Hirschsprung's disease	No ☐	Yes ☐	

Tracheoesophageal fistula	No ☐	Yes ☐		
Esophageal obstruction	No ☐	Yes ☐		
Intestinal atresia	No ☐	Yes ☐		
Anal atresia	No ☐	Yes ☐		
Anteriorly placed anus	No ☐	Yes ☐		
Umbilical hernia	No ☐	Yes ☐		
other				
**Psychomotor and intellectual development**				
Developmental delay	No ☐	Yes ☐		
Language delay	No ☐	Yes ☐		
Intellectual disability	No ☐	Yes ☐	☐ borderline<br>☐ mild<br>☐ moderate<br>☐ severe<br>☐ deep	
**Learning difficulties and neuropsychological manifestations**				
Attention difficulties	No ☐	Yes ☐		
Visual spatial difficulties	No ☐	Yes ☐		
Problems with understanding abstract concepts	No ☐	Yes ☐		
Impaired executive function	No ☐	Yes ☐		
Non-verbal learning disorder	No ☐	Yes ☐		
ADHD	No ☐	Yes ☐		
Autism spectrum disorders	No ☐	Yes ☐		
other				
**Psychiatric/behaviour problems**				
Schizophrenia	No ☐	Yes ☐		
Anxiety disorders	No ☐	Yes ☐		
Phobias	No ☐	Yes ☐		
Psychotic disorders	No ☐	Yes ☐		
Obsessive-compulsive disorders	No ☐	Yes ☐		
Depression	No ☐	Yes ☐		
Rapid mood changes	No ☐	Yes ☐		
Atypical social interaction skills	No ☐	Yes ☐		
other				
**Other**				
Supernumerary spleen	No ☐	Yes ☐		
Diaphragmatic hernia	No ☐	Yes ☐		
Bernard-Soulier Syndrome	No ☐	Yes ☐		
Hepatoblastoma	No ☐	Yes ☐		

Neuroblastoma	No ☐	Yes ☐	
Early-onset Parkinson's disease	No ☐	Yes ☐	
Wilms' tumor	No ☐	Yes ☐	
Rhabdomyosarcoma	No ☐	Yes ☐	
other			
Special skills (e.g. musical talent)	No ☐	Yes ☐	
other			

Prenatal ultrasound findings in patient

	NO	YES	COMMENTS
Cardiac anomalies	No ☐	Yes ☐	☐ Interrupted aortic arch, IAA<br>☐ Persistent truncus arteriosus, PTA<br>☐ Tetralogy of Fallot<br>☐ Ventricular septal defect, VSD<br>☐ other
Echogenic intracardiac focus, EIF	No ☐	Yes ☐	
Increased NT	No ☐	Yes ☐	
IUGR	No ☐	Yes ☐	
Cleft palate	No ☐	Yes ☐	☐ bilateral<br>☐ unilateral
Cleft lip	No ☐	Yes ☐	☐ bilateral<br>☐ unilateral
Polyhydramnios	No ☐	Yes ☐	
Oligohydramnios	No ☐	Yes ☐	
Ventriculomegaly	No ☐	Yes ☐	
Enlarged cisterna magna	No ☐	Yes ☐	
Choroid plexus cysts	No ☐	Yes ☐	
Meningomyelocele	No ☐	Yes ☐	
Craniosynostosis	No ☐	Yes ☐	
Spina bifida	No ☐	Yes ☐	
Polidaktyly	No ☐	Yes ☐	
Clubfoot	No ☐	Yes ☐	
Diaphragmatic hernia	No ☐	Yes ☐	
2-vessel cord	No ☐	Yes ☐	
Absent renal artery	No ☐	Yes ☐	
Renal pelvis dilatation	No ☐	Yes ☐	
Abnormal kidney echogenicity	No ☐	Yes ☐	
Polycystic kidney	No ☐	Yes ☐	
Urinary obstruction	No ☐	Yes ☐	
Inguinal hernia	No ☐	Yes ☐	
Echogenic bowel	No ☐	Yes ☐	
Gastroschisis	No ☐	Yes ☐	
Decreased fetal movements	No ☐	Yes ☐	
Placental insufficiency	No ☐	Yes ☐	
other			
