# Supplementary material for: Coexisting Conditions Modifying Phenotypes of Patients with 22q11.2 Deletion Syndrome
Source: Genes (Basel). 2023 Mar 9;14(3):680. doi: 10.3390/genes14030680 (PMC10048180; doi:10.3390/genes14030680)
Supplement: Supplementary file 1 [file genes-14-00680-s001.zip › Supplementary Table S3.pdf]

Table S3. Hemizygotic variants in the 22q11.2 deletion region.

Identifier	Sex	Age	key	Gene	Exonic Function	Change	gnomAD exome ALL
GC034781	M	4 y	chr22:19972989_A>G	ARVCF	nonsynonymous SNV	NM_001670:c.T2486C:p.V829A	9e-04
GC034782	M	3 y	chr22:19408570_T>C	HIRA	nonsynonymous SNV	NM_003325:c.A124G:p.I42V	4.063e-06
GC034783	F	8 y	chr22:20489028_C>G	KLHL22	nonsynonymous SNV	NM_032775:c.G184C:p.E62Q	8e-04
GC034784	M	15 y	chr22:18606898_G>A	RIMBP3	nonsynonymous SNV	NM_015672:c.C4537T:p.R1513C	4e-04
GC034784	M	15 y	chr22:20112643_->C	TRMT2A	frameshift insertion	NM_022727:c.1797dupG:p.P600fs	0.0086
GC034784	M	15 y	chr22:20405601_C>G	ZNF74	nonsynonymous SNV	NM_001256523:c.C482G:p.T161R	7e-04
GC034788	M	2 y	chr22:19209093_T>G	CLTCL1	nonsynonymous SNV	NM_001835:c.A3271C:p.N1091H	1e-04
GC034788	M	2 y	chr22:19851751_A>G	C22orf29	nonsynonymous SNV	NM_024627:c.T511C:p.F171L	7e-04
GC034788	M	2 y	chr22:19851895_C>A	C22orf29	nonsynonymous SNV	NM_024627:c.G367T:p.D123Y	0.007
GC034789	F	6 m	chr22:19435879_C>T	MRPL40	nonsynonymous SNV	NM_001318152:c.C406T:p.P136S	0
GC034789	F	6 m	chr22:20858678_->CCGCCT	PI4KA	nonframeshift insertion	NM_058004:c.47_48insAGGCGG:p.G16delinsGGG	0.004
GC034790	F	33 y	chr22:19974169_G>C	ARVCF	nonsynonymous SNV	NM_001670:c.C2031G:p.F677L	0.0026
GC034790	F	33 y	chr22:20112643_->C	TRMT2A	frameshift insertion	NM_022727:c.1797dupG:p.P600fs	0.0086
GC034791	M	26 y	chr22:21030186_A>C	SLC7A4	nonsynonymous SNV	NM_004173:c.T1148G:p.L383R	0.0029
GC034792	F	6 y	chr22:20976529_G>A	AIFM3	nonsynonymous SNV	NM_001018060:c.G1021A:p.G341S	0.002
GC034822	M	10 y	chr22:21052293_G>A	LRRC74B	nonsynonymous SNV	NM_001291006:c.G667A:p.E223K	0.0018
GC034836	M	6 y	chr22:20779543_C>A	SERPIND1	nonsynonymous SNV	NM_000185:c.C231A:p.D77E	0.0068
GC034850	M	3 m	chr22:19979026_T>C	ARVCF	nonsynonymous SNV	NM_001670:c.A1451G:p.H484R	8.952e-05
GC034850	M	3 m	chr22:20779331_G>A	SERPIND1	nonsynonymous SNV	NM_000185:c.G19A:p.A7T	0.0072
GC034872	M	10 y	chr22:20918068_C>T	CRKL	nonsynonymous SNV	NM_005207:c.C134T:p.P45L	8.122e-06
GC034872	M	10 y	chr22:21386970_G>A	RIMBP3B,RIMBP3C	nonsynonymous SNV	NM_001128633:c.G3112A:p.V1038M	0
GC034873	M	1 y 4 m	chr22:19972989_A>G	ARVCF	nonsynonymous SNV	NM_001670:c.T2486C:p.V829A	9e-04
GC028958	F	2 weeks	chr22:19505408_C>T	CDC45	nonsynonymous SNV	NM_001178011:c.C613T:p.R205C	2,03E-05
GC034898	F	9 m	chr22:18606898_G>A	RIMBP3	nonsynonymous SNV	NM_015672:c.C4537T:p.R1513C	4e-04
GC034926	F	1 y 4 m	chr22:20143362_G>A	ZDHHC8	nonsynonymous SNV	NM_001185024:c.G1732A:p.V578I	7,00E-04
GC034929	M	4 y	chr22:21060419_G>A	LRRC74B	nonsynonymous SNV	NM_001291006:c.G1070A:p.R357K	6e-04
GC034933	F	1 y	chr22:21031247_C>T	SLC7A4	nonsynonymous SNV	NM_004173:c.G566A:p.R189H	3.259e-05
GC034945	F	27 y	chr22:19981313_G>A	ARVCF	nonsynonymous SNV	NM_001670:c.C794T:p.T265M	4.218e-05
GC034947	M	1 m	chr22:18606898_G>A	RIMBP3	nonsynonymous SNV	NM_015672:c.C4537T:p.R1513C	4e-04
