# Supplementary material for: Coexisting Conditions Modifying Phenotypes of Patients with 22q11.2 Deletion Syndrome
Source: Genes (Basel). 2023 Mar 9;14(3):680. doi: 10.3390/genes14030680 (PMC10048180; doi:10.3390/genes14030680)
Supplement: Supplementary file 1 [file genes-14-00680-s001.zip › Supplementary Table S4.pdf]

Table S4. ClinVar pathogenic cancer predisposition.

Identifier	Chr	Start	End	Change	Zyg	Gene	OMIM diseases	Exonic Function	gnomAD exome ALL
GC030953	chr11	108312424	108312424	NM_000051:c.G5932T:p.E1978X	het	*ATM*	Ataxia-telangiectasia;<br>Lymphoma, B-cell non-Hodgkin, somatic;<br>Lymphoma, mantle cell, somatic;<br>T-cell prolymphocytic leukemia, somatic;<br>Breast cancer, susceptibility to	stopgain	4.877e-05
GC034800	chr17	43057062	43057062	NM_007297:c.5125dupC:p.Q1709fs	het	*BRCA1*	Fanconi anemia, complementation group S;<br>Breast-ovarian cancer, familial, 1;<br>Pancreatic cancer, susceptibility to, 4	frameshift insertion	2e-04
GC034899	chr17	43057062	43057062	NM_007297:c.5125dupC:p.Q1709fs	het	*BRCA1*	Fanconi anemia, complementation group S;<br>Breast-ovarian cancer, familial, 1;<br>Pancreatic cancer, susceptibility to, 4	frameshift insertion	2e-04
GC034772	chr13	32333272	32333276	NM_000059:c.1794_1798del:p.T598fs	het	*BRCA2*	Fanconi anemia, complementation group D1;<br>Wilms tumor;<br>Breast cancer, male, susceptibility to;<br>Breast-ovarian cancer, familial, 2;<br>Glioblastoma 3;<br>Medulloblastoma;<br>Pancreatic cancer 2;<br>Prostate cancer	frameshift deletion	4.182e-06
GC034784	chr13	32338329	32338329	NM_000059:c.3974_3975insTGCT:p.T1325fs	het	*BRCA2*	Fanconi anemia, complementation group D1;<br>Wilms tumor;<br>Breast cancer, male, susceptibility to;<br>Breast-ovarian cancer, familial, 2;<br>Glioblastoma 3;<br>Medulloblastoma;<br>Pancreatic cancer 2;<br>Prostate cancer	frameshift insertion	4.444e-06
